# Constructing a clinical prediction model for patients with coronary heart disease: a study based on carotid ultrasound and clinical related factors

**DOI:** 10.3389/fcvm.2025.1676831

**Published:** 2025-11-25

**Authors:** Pingjuan Ni, Wenrong Wang, Nan Wang, Pinjing Hui

**Affiliations:** 1 Department of Neurosurgery, The First Affiliated Hospital of Soochow University, Suzhou, Jiangsu, China; 2 Department of Stroke Center, The First Affiliated Hospital of Soochow University, Suzhou, Jiangsu, China; 3 Department of Ultrasound, The Second Hospital of Shandong University, Jinan, Shandong, China; 4 Department of Ultrasound, Lianyungang Maternal and Child Health Hospital, Lianyungang, Jiangsu, China

**Keywords:** coronary heart disease, ultrasound, carotid plaques, prediction model, nomogram

## Abstract

**Background and objective:**

The incidence of coronary heart disease(CHD) is high and its onset is insidious. CHD often occurs due to the formation of atherosclerotic plaques in the coronary arteries, which leads to narrowing or even occlusion of the lumen, and is often accompanied by the formation of atherosclerotic plaques in the carotid arteries. This study aims to construct a prediction model for patients with CHD by evaluating the carotid ultrasound(CDU) and integrating clinical related factors.

**Methods:**

A total of 649 patients were enrolled and divided into the CHD and the non-CHD groups according to coronary angiography (CAG) results. CDU assessment was performed 24 h before CAG, and basic patient information and clinical related factors were recorded. Patients were randomly divided into training and validation sets in a 7:3 ratio. In the training set, univariate and multivariate logistic regression analyses were used to screen independent risk factors for patients with CHD, and a risk prediction model was constructed based on these factors. The model was validated using the receiver operating characteristic and calibration curves, decision curve analysis, and clinical impact curve.

**Results:**

Multivariate logistic regression analysis of the training set exhibited that male chest pain, diabetes, triglyceride, Apolipoprotein A, plaque score, and carotid stenosis were independent predictors of CHD (*p* < 0.05). The nomogram constructed using these indicators exhibited high predictive performance, calibration, and clinical value in training and validation sets.

**Conclusion:**

The CHD prediction model constructed by CDU combined with clinical related factors is simple, practical, and has stable prediction performance, which can stratify suspicious patients and further guide clinical diagnosis and treatment.

## 1 Introduction

According to the European Society of Cardiology (ESC) statistics for 2021 (1), cardiovascular disease is the most common cause of death in ESC member states, with the total number of deaths far exceeding cancer deaths. Among them, coronary heart disease (CHD) is myocardial ischemia caused by a narrowing or blockage of the coronary arteries. CHD incidence continues to rise due to the fast pace of life, unhealthy diet, high work pressure, and lack of physical exercise in modern society (2). In 2022, the number of patients with cardiovascular disease in China was about 330 million, of which 11.39 million had CHD (3). CHD has become a major social health problem that seriously harms human physical and mental health and increases the social and economic burden. Therefore, CHD diagnosis and prevention have become the focus and hot spot of the current research of cardiologists and sonographers. The main tests for CHD include an electrocardiogram (ECG), echocardiography, coronary computed tomography angiography (CCTA), and coronary angiography (CAG). Among them, CAG is the gold standard for CHD diagnosis, which can determine whether coronary artery stenosis exists, as well as the location, degree, and scope of stenosis (4). In the early stage of CHD, when the lesion is mild and the clinical symptoms are not typical, ECG and echocardiography often lack specificity and are difficult to diagnose. CCTA and CAG are relatively mature, but they are more expensive, involve radiation exposure, and are more complex to operate. Additionally, CAG has complications such as dissection aneurysm, malignant arrhythmia, and kidney injury (5), which are often not easily accepted by patients; therefore, routine implementation is difficult and not suitable for early screening of CHD. As a result, clinical practice lacks a completely non-invasive tool for early assessment of CHD risk.

The heart is our blood-pumping organ, supplying the myocardium through the left and right coronary arteries on the one hand and the intracranial through the aortic arch through the carotid arteries, vertebral arteries, and subclavian arteries on the other. If atherosclerosis occurs in the coronary arteries, severe cases will cause myocardial infarction; if atherosclerosis occurs in the carotid arteries, severe cases will cause cerebral infarction. The risk factors, pathogenesis, and hemodynamics of both are similar and often go hand-in-hand (6, 7). The carotid artery is located superficially, and ultrasound can be used to observe the vessel wall and vessel lumen in cross and longitudinal sections in real-time, which is non-invasive and convenient. Theoretically, it can be used as a window to observe the coronary arteries. Previous studies have indicated that ultrasound can assist in predicting the presence and severity of coronary artery disease by assessing the degree of carotid atherosclerosis (8–11). However, most of the existing literature has focused on intima-media thickness (IMT) (12), maximum carotid plaque height (MPH), maximum plaque length, and total plaque area (TPA) (7, 9, 13, 14). The predictive role of IMT in cardiovascular disease is currently controversial, and a growing number of studies have exhibited that ultrasound assessment of carotid plaque is more accurate in cardiovascular disease diagnosis than IMT (15). However, due to the time-consuming nature of plaque measurement and calculation, it is difficult to implement in general clinical practice. For this reason, the American Society of Echocardiography recommends the Rotterdam method to calculate the plaque score (PS) (16), which can be obtained with only 4–6 ultrasound images, which is simple and fast. Studies have indicated that carotid PS is associated with ischemic stroke and major adverse cardiovascular events (17, 18). This study used the neck as an observation window to measure PS based on carotid ultrasound (CDU), combined with other clinical indicators, to construct a non-invasive risk prediction model for CHD to provide personalized stratified diagnosis and prevention for patients.

## 2 Materials and methods

### 2.1 Participants and study design

**Figure 1:**
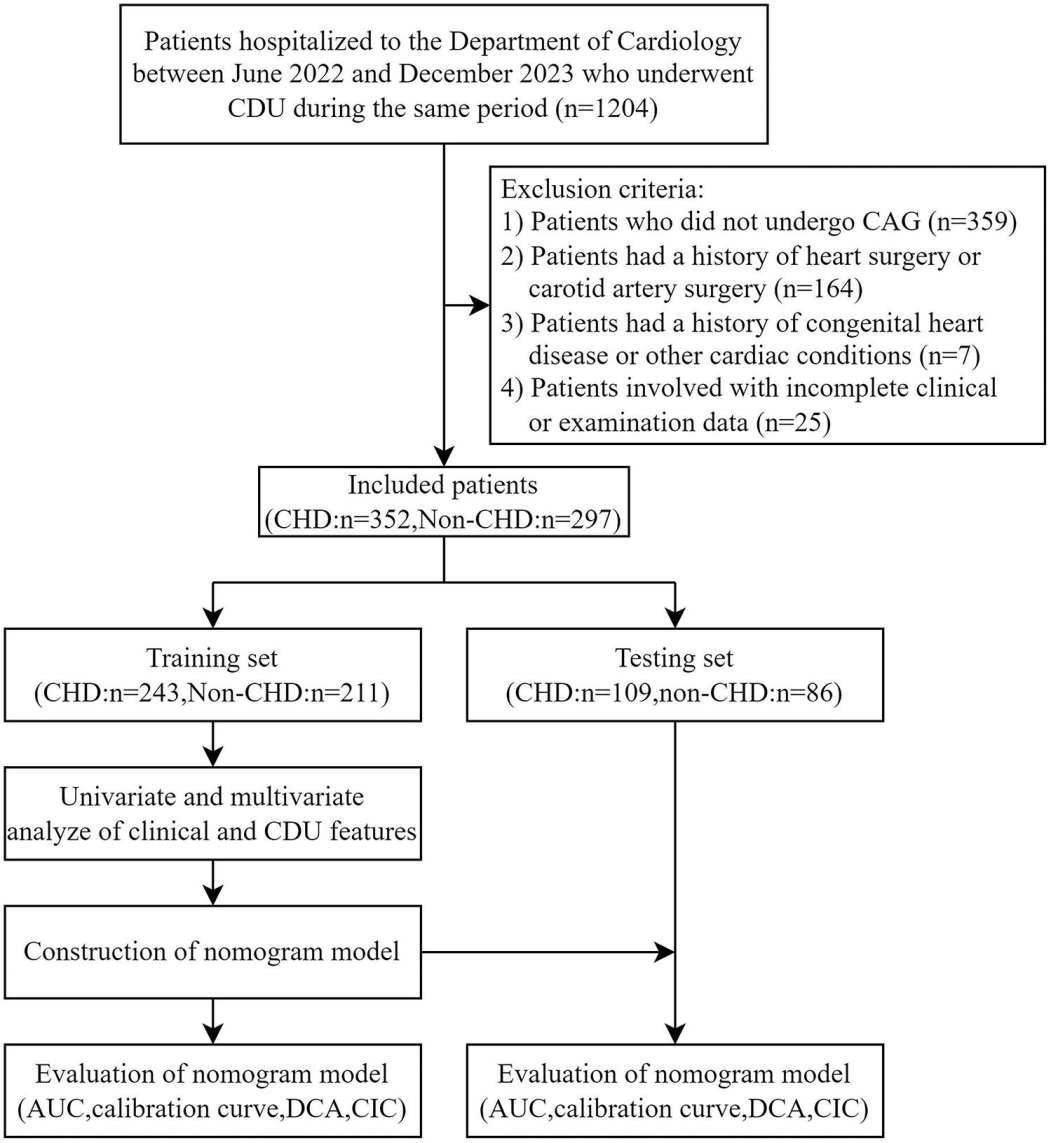
Flowchart of this study.

Patients with suspected CHD admitted to the Department of Cardiology in our hospital from July 2022 to December 2023 and receiving CDU during the same period were recruited. The exclusion criteria were as follows: (1) Patients who did not undergo CAG for some reason; (2) patients with a previous history of heart or carotid artery surgery; (3) patients with congenital heart disease or other heart diseases; (4) patients with incomplete clinical data or examination data. A total of 649 patients were enrolled in the study, including 352 in the CHD group and 297 in the non-CHD group. After randomization at a ratio of 7:3, 454 patients entered the training set, and 195 patients entered the validation set. CDU images were collected from ultrasound workstations, and clinical information and CAG data were obtained from the inpatient electronic medical record system. The flowchart of this study is illustrated in Figure 1.

### 2.2 Clinical data collection

In basic clinical information of a patient, body mass index (BMI) was calculated as weight (kg) divided by the square of height (m^2^). Smoking was defined as at least one cigarette per day for over a year (19). Hypertension was defined as having a history of high blood pressure, currently taking antihypertensive medications, or two consecutive resting systolic blood pressure ≥140 mmHg and/or diastolic blood pressure ≥90 mmHg without antihypertensive medications. Diabetes was defined as patients with fasting blood glucose levels ≥7.0 mmol/L, random blood glucose ≥11.1 mmol/L, or glycosylated hemoglobin ≥6.5%. Laboratory indicators also included total cholesterol (TC), triglycerides (TG), low-density lipoprotein cholesterol (LDL-C), high-density lipoprotein cholesterol (HDL-C), Apolipoprotein A (Apo A), Apolipoprotein B (Apo B), lipoprotein a (Lip a), hypersensitive C-reactive protein (Hs-CRP), and others.

### 2.3 Ultrasonic examination

**Figure 2:**
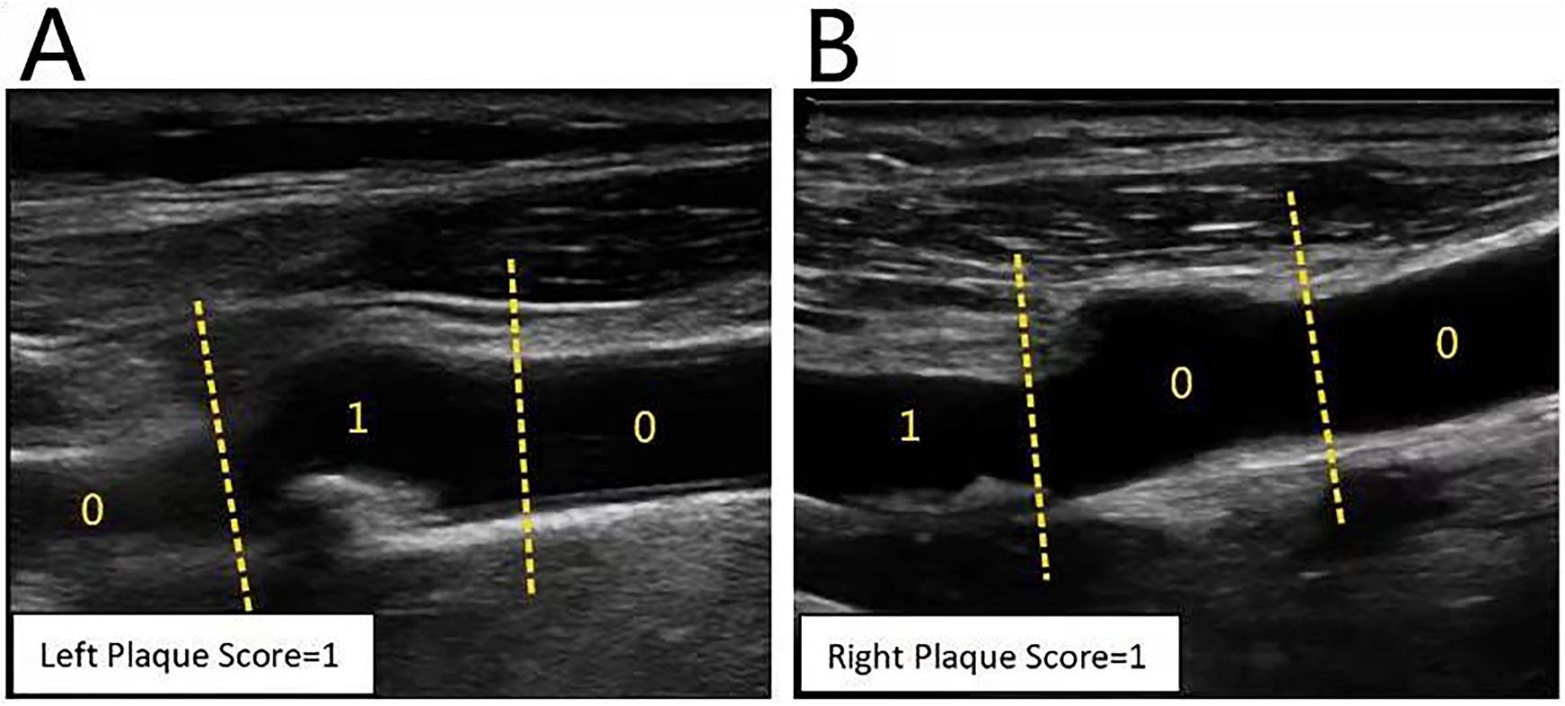
Calculation of carotid plaque score. **(A)** CDU showed the left plaque score was 1[0(for CCA) + 1(for Bulb) + 0(for ICA)]; **(B)** CDU showed the right plaque score was 1[0(for CCA) + 0(for Bulb) + 1(for ICA)]; So this patient's carotid plaque score was 2.

Philips iU Elite color Doppler ultrasonic diagnosis instrument (Philips, Netherlands) was used, and an L9–3 linear array probe was selected with a frequency of 3–9 MHz. The carotid artery was continuously scanned in cross and longitudinal sections, respectively. Specifically, starting from the common carotid artery (CCA), moving toward the bulb, and scanning along the internal carotid artery (ICA) to observe plaque and other lesions, the location, size, shape, fibrous cap, and other characteristics of the plaque were recorded. The Rotterdam method (16) was used to calculate PS based on bilateral CCA, Bulb, and ICA segments. If there was a plaque in a certain segment, it was counted as 1 point. The minimum score was “0” and the maximum score was “6”. The score requires only the presence or absence of plaques and does not consider the size or number of plaques (Figure 2).

The CDU assessed the degree of carotid artery stenosis according to the North American Symptomatic Carotid Endarterectomy Trial (20). No stenosis was defined as normal diameter without stenosis; mild stenosis was defined as a stenosis rate between 0% and 49%; moderate stenosis was defined as a stenosis rate between 50% and 69%, and severe stenosis was defined as a stenosis rate between 70% and 99%. Vulnerable plaques were characterized as heterogeneous, hypoechoic plaques with an irregular morphology, incomplete fibrous cap, or intraplaque blood flow signals on CDU (21), as well as surface and multiple calcifications, and ulceration of atherosclerotic plaques (22).

### 2.4 CAG examination

An experienced cardiovascular physician evaluated the results. The CHD group was defined as one or more major coronary arteries (left main artery, left anterior descending branch, left circumflex branch, right coronary artery) with ≥50% stenosis (23), while the non-CHD group was defined as <50% stenosis or no stenosis.

### 2.5 Statistical analysis

Statistical Package for Social Science software (Version 20.0) was used for data analysis. Continuous variables conforming to a normal distribution are expressed as mean ± standard deviation and were analyzed using Student's *t*-test. Continuous variables not conforming to a normal distribution are represented by the median (P25, P75) and were tested by the Mann–Whitney *U* test. Missing data were minimal, with no variable having more than 5% missingness. Therefore, a complete-case analysis was performed, and only participants with complete data were included in the final model. Logistic regression was used for univariate and multivariate analyses. Multicollinearity among the independent variables was assessed using the variance inflation factor(VIF).A value lower than 5 was considered to indicate no severe multicollinearity. Restricted cubic splines(RCS) were used to analyze the nonlinear relationship between continuous variables and the likelihood of CHD. The R software package (Version 4.2.1) was used to construct the nomo prediction model. The model was evaluated by receiver operating characteristic curve (ROC), calibration curve, decision curve analysis (DCA), and clinical impact curve (CIC). To evaluate model overfitting and assess the statistical stability of the performance estimate, we performed an internal validation using bootstrap resampling with 1,000 repetitions. For each bootstrap sample, the model was refitted, and the optimism was calculated by comparing its performance on the bootstrap sample to that on the original dataset. We report the optimism-corrected AUC and its 95% confidence interval (CI),derived from the distribution of the 1,000 bootstrap estimates. Statistical significance was set at *p* < 0.05.

## 3 Results

### 3.1 Clinical and CDU characteristics of patients

In total, 649 patients met the inclusion criteria, including 352 patients with CHD and 297 patients without CHD. The detailed clinical and CDU characteristics are presented in Table 1. According to a 7:3 ratio, 454 patients were randomly assigned to the training set, and 195 were randomly assigned to the validation set. There were no significant differences in baseline characteristics between the two datasets (*p* > 0.05) (Table 1).

**Table 1:** The baseline characteristics of the enrolled patients.

Variables	Total (*n* = 649)	Training set (*n* = 454)	Testing set (*n* = 195)	*p*	CHD (*n* = 352)	Non-CHD (*n* = 297)	*p*
Age, years	66.00 (58.00,72.00)	65.00 (58.00,72.00)	66.00 (58.00,72.00)	0.875	65.00 (58.00,72.00)	66.00 (58.00;72.00)	0.506
Male (*n*, %)	358 (55.16%)	246 (54.19%)	112 (57.44%)	0.498	235 (66.76%)	123 (41.41%)	<0.001
BMI	24.46 (22.83,26.42)	24.52 (22.83,26.35)	24.30 (22.80,26.56)	0.806	24.50 (22.90,26.83)	24.35 (22.65,26.17)	0.102
Chest pain (*n*, %)	568 (87.52%)	398 (87.67%)	170 (87.18%)	0.966	322 (91.48%)	246 (82.83%)	0.001
Current smoke (*n*, %)	143 (22.03%)	98 (21.59%)	45 (23.08%)	0.751	103 (29.26%)	40 (13.47%)	<0.001
Hypertension (*n*, %)	466 (71.80%)	331 (72.91%)	135 (69.23%)	0.390	270 (76.70%)	196 (65.99%)	0.003
Diabetes (*n*, %)	256 (39.45%)	175 (38.55%)	81 (41.54%)	0.530	164 (46.59%)	92 (30.98%)	<0.001
LDL-C	2.25 (1.73,2.96)	2.22 (1.71,2.92)	2.35 (1.75,3.04)	0.436	2.21 (1.62,2.98)	2.34 (1.77,2.94)	0.305
HDL-C	1.00 (0.85,1.22)	1.01 (0.84,1.23)	0.99 (0.88,1.19)	0.984	0.94 (0.80,1.12)	1.07 (0.92,1.29)	<0.001
TG	1.31 (1.00,1.77)	1.31 (1.01,1.71)	1.29 (0.92,1.89)	0.988	1.38 (1.02,1.91)	1.25 (0.95,1.60)	0.004
TC	4.02 (3.40,4.79)	3.99 (3.36,4.76)	4.10 (3.46,4.86)	0.285	4.01 (3.31,4.80)	4.03 (3.54,4.77)	0.176
Apo A	1.16 (1.04,1.30)	1.16 (1.03,1.31)	1.16 (1.05,1.27)	0.935	1.12 (0.99,1.24)	1.20 (1.08,1.36)	<0.001
Apo B	0.70 (0.58,0.88)	0.70 (0.59,0.87)	0.71 (0.58,0.89)	0.677	0.70 (0.58,0.88)	0.71 (0.58,0.86)	0.635
Lip a	149.10 (85.10,277.70)	149.10 (84.62,283.83)	149.10 (86.25,247.70)	0.764	148.85 (77.57,286.78)	149.10 (90.80,267.80)	0.667
Hs-CRP	1.84 (0.73,4.92)	1.78 (0.73,4.73)	1.97 (0.74,5.72)	0.424	1.92 (0.80,5.21)	1.59 (0.71,4.84)	0.117
LVEF	63.00 (59.00,66.00)	63.00 (59.00,66.00)	63.00 (59.00,66.00)	0.902	62.00 (58.00,66.00)	63.00 (60.00,66.00)	0.007
MPH	1.90 (0.00,2.70)	1.90 (0.00,2.70)	1.90 (0.00,2.67)	0.850	2.20 (1.60,3.00)	1.60 (0.00,2.20)	<0.001
Vulnerable plaque (*n*, %)	240 (36.98%)	171 (37.67%)	69 (35.38%)	0.643	161 (45.74%)	79 (26.60%)	<0.001
Carotid stenosis (*n*, %)	435 (67.03%)	308 (67.84%)	127 (65.13%)	0.677	272 (77.27%)	163 (54.88%)	<0.001
Absent	214	146	68	-	80	134	-
Low	408	291	117	-	250	158	-
Moderate	13	8	5	-	10	3	-
Severe	14	9	5	-	12	2	-
Plaque score	1 (0,3)	1 (0,3)	1 (0,2)	0.557	2 (1,3)	1 (0,2)	<0.001

### 3.2 Univariate and multivariate analyses of the training set

**Figure 3:**
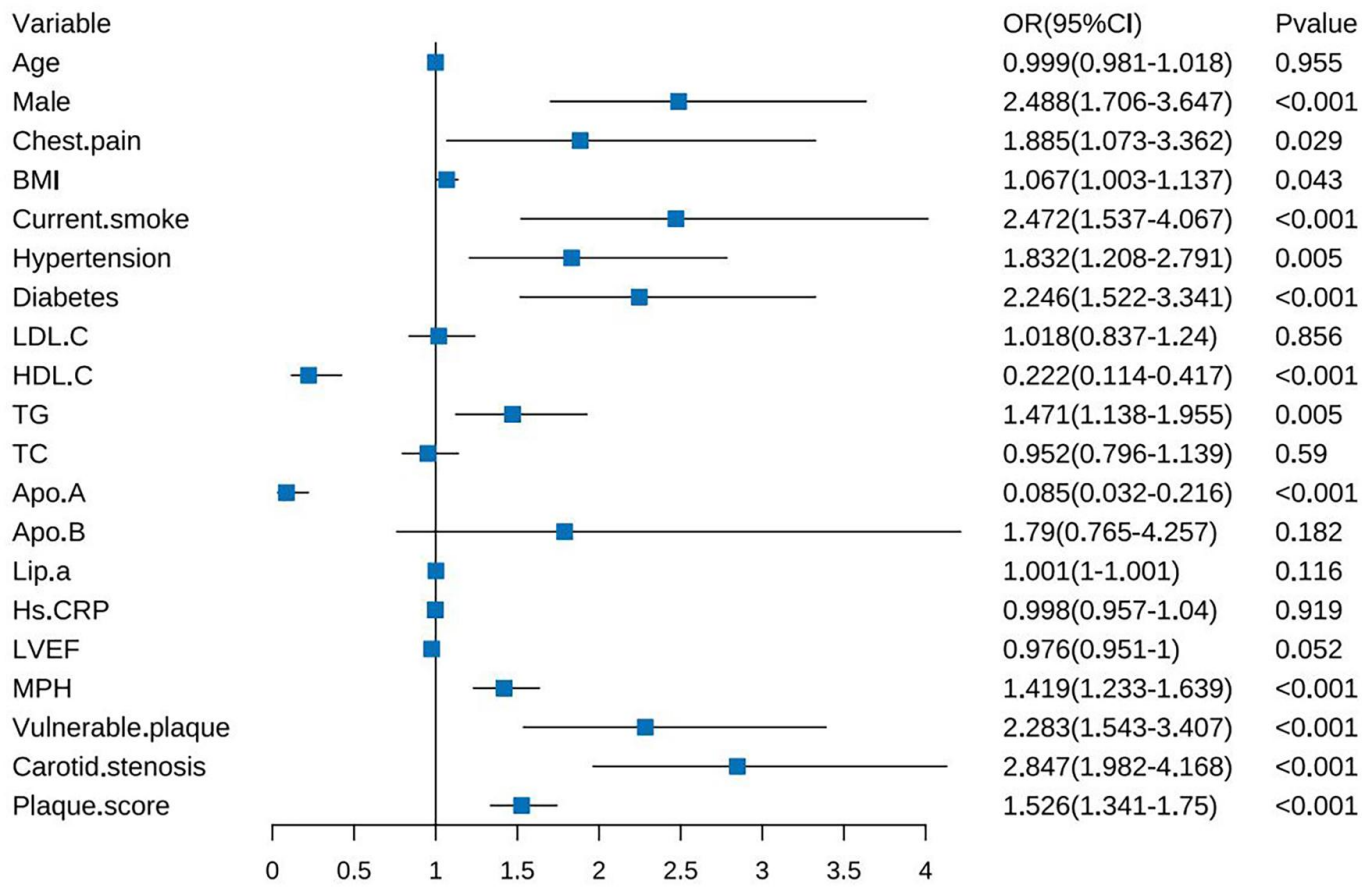
Univariate logistic regression analysis in the training set.

Univariate logistic regression analysis was performed on the training set, and statistical differences were found in some clinical features (male, chest pain, BMI, smoking, hypertension, diabetes, HDL-C, TG, and Apo A) and some CDU features (MPH, vulnerable plaque, carotid stenosis, and PS) (*p* < 0.05), as illustrated in Forest Figure 3. These indicators were included in multivariate logistic regression, and the results exhibited that male, chest pain, diabetes, TG, Apo A, PS, and carotid stenosis were independent predictors of CHD (*p* *<* 0.05). The detailed results of the regression analysis, including regression coefficients,odds ratio(OR), CI, and *p*-values,were presented in Table 2.

**Table 2:** Multivariate logistic regression analysis and VIF in the training set.

Variables	B	OR	95%CI		*P*	VIF
			Lower limit	Upper limit		
Intercept	−0.453	–	–	–	0.585	–
Male	0.554	1.739	1.127	2.686	0.012	1.096
Chest pain	0.946	2.576	1.365	4.960	0.004	1.044
Diabetes	0.605	1.832	1.178	2.863	0.007	1.052
TG	0.360	1.433	1.074	1.962	0.019	1.011
Apo A	−1.704	0.182	0.061	0.524	0.002	1.111
Plaque score	0.229	1.257	1.031	1.546	0.026	2.154
Carotid stenosis	0.597	1.817	1.052	3.205	0.035	2.150

### 3.3 Construction and verification of the CHD prediction model

**Figure 4:**
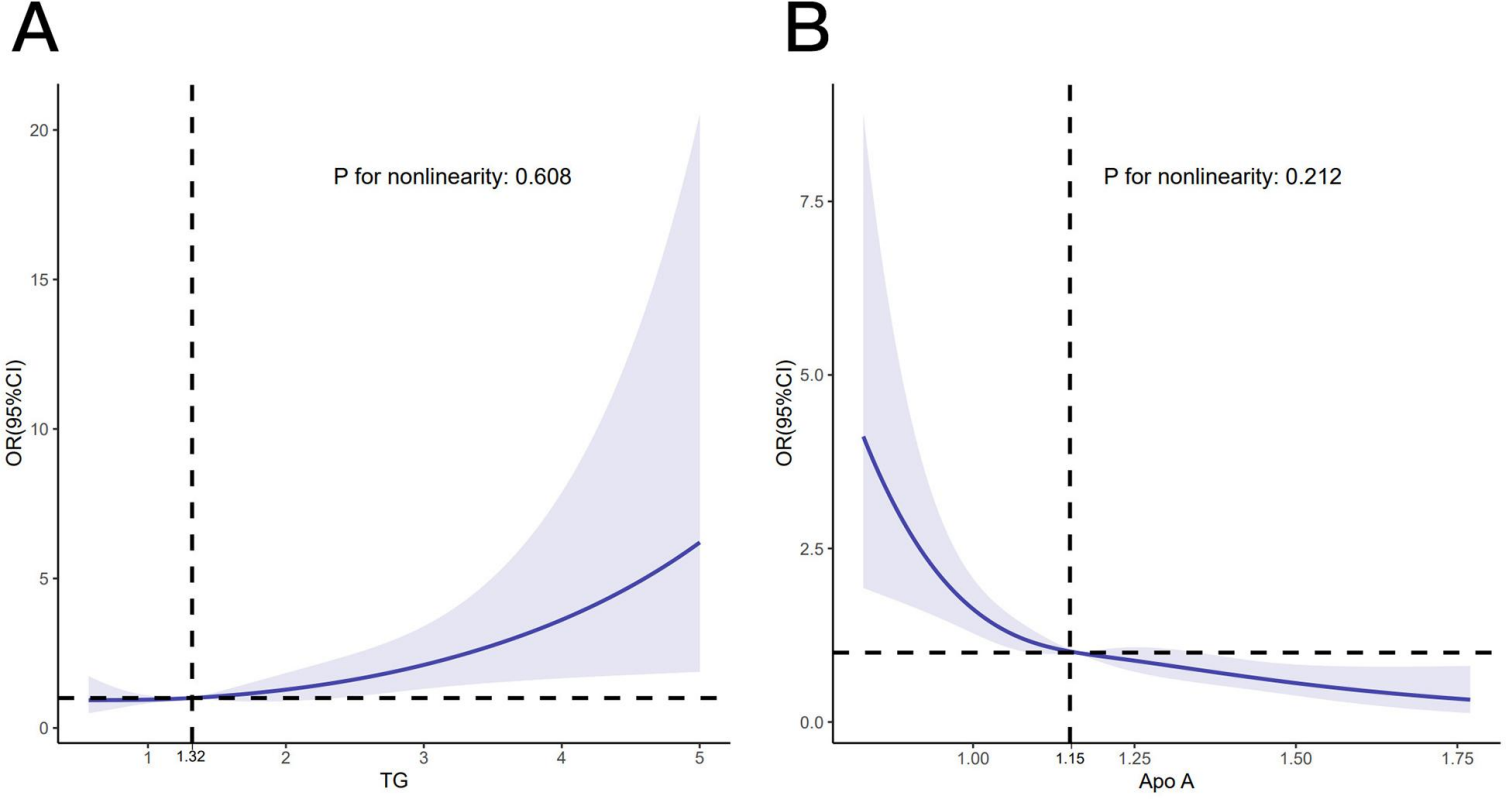
RCS curve. RCS curve displaying the relationship between TG **(A)** and Apo A **(B)** with the proablity of CHD.

Indicators with statistical differences presented by multivariate logistic regression in the training set were used to construct a nomo model to predict CHD probability in patients. The VIF was examined to assess multicollinearity among the predictors. All VIF values were below 5 (range: 1.011–2.154) (Table 2), confirming that multicollinearity did not pose a significant issue to the model. The relationship of the continuous variables (TG and Apo A) with CHD was evaluated using RCS (Figure 4). Since the likelihood ratio test for nonlinearity was not significant (*p* = 0.608 and 0.212, respectively) and the spline plots demonstrated linearity, these variables were incorporated as linear terms in the final model.

**Figure 5:**
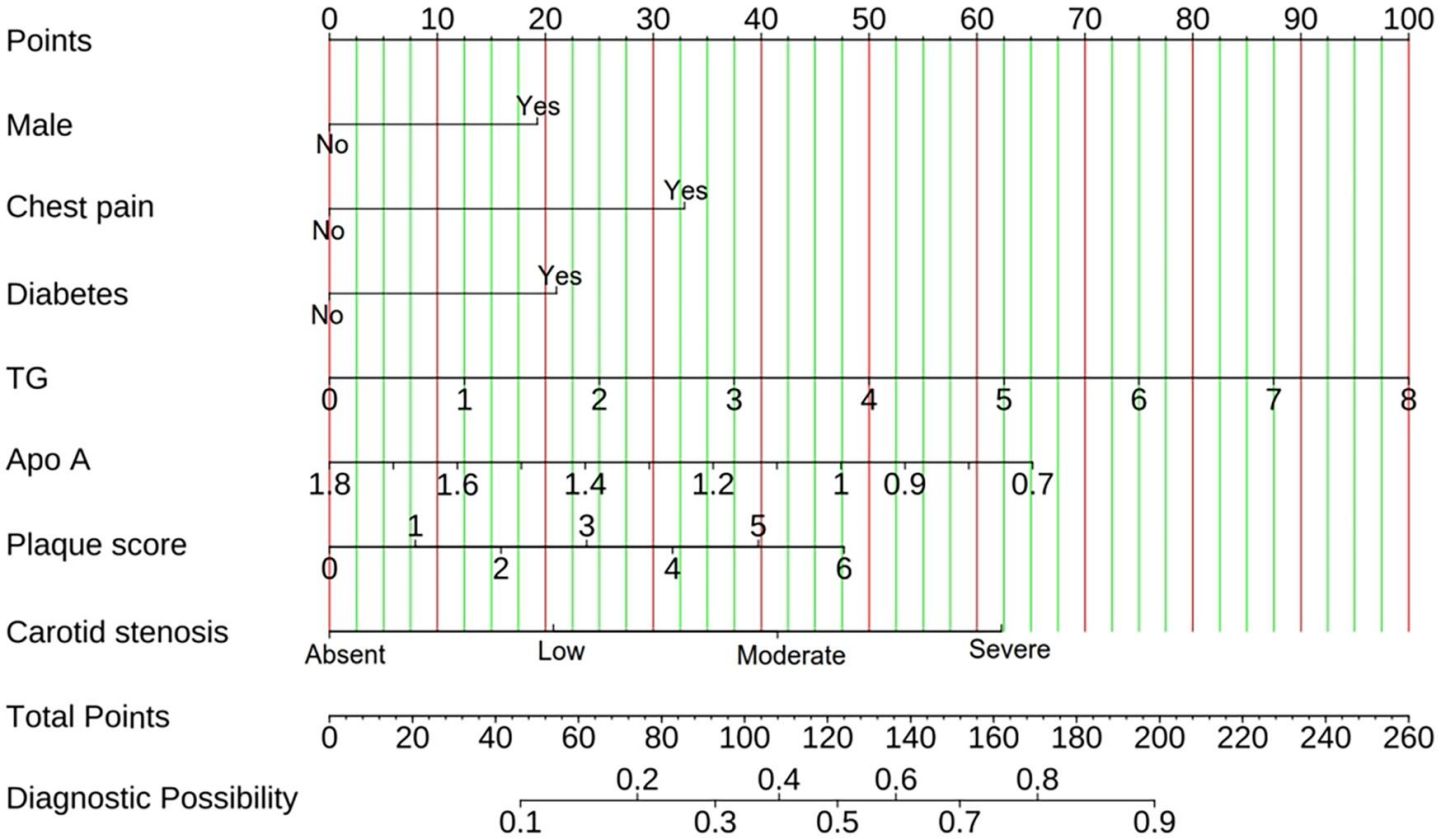
Nomogram model for predicting CHD.

Based on the regression coefficients in Table 2, a prediction formula for calculating the individual risk of CHD was constructed as follows: Logit(*p*) = −0.453 + (0.554 × Male) + (0.946 × Chest pain) + (0.605 × Diabetes) + (0.360 × TG) − (1.704 ×Apo A) + (0.229×Plaque score) + (0.597 × Carotid stenosis),where *p* represents the probability of CHD. In this formula, binary variables (male, chest pain, diabetes) were assigned as “yes = 1, no = 0”;continuous variables(TG in mmol/L, Apo A in g/L) were included in their original measured values;plaque score(0–6 points) and carotid stenosis(no = 0, mild = 1, moderate = 2, severe = 3) were treated as ordered variables. To make this predictive model more user-friendly in clinical practice, we constructed a nomogram based on the above formula (Figure 5). As illustrated in the Figure 5, each indicator of each patient corresponds to a certain score, and the total score can be obtained by adding them together, and then the CHD risk for each patient can be determined.

**Figure 6:**
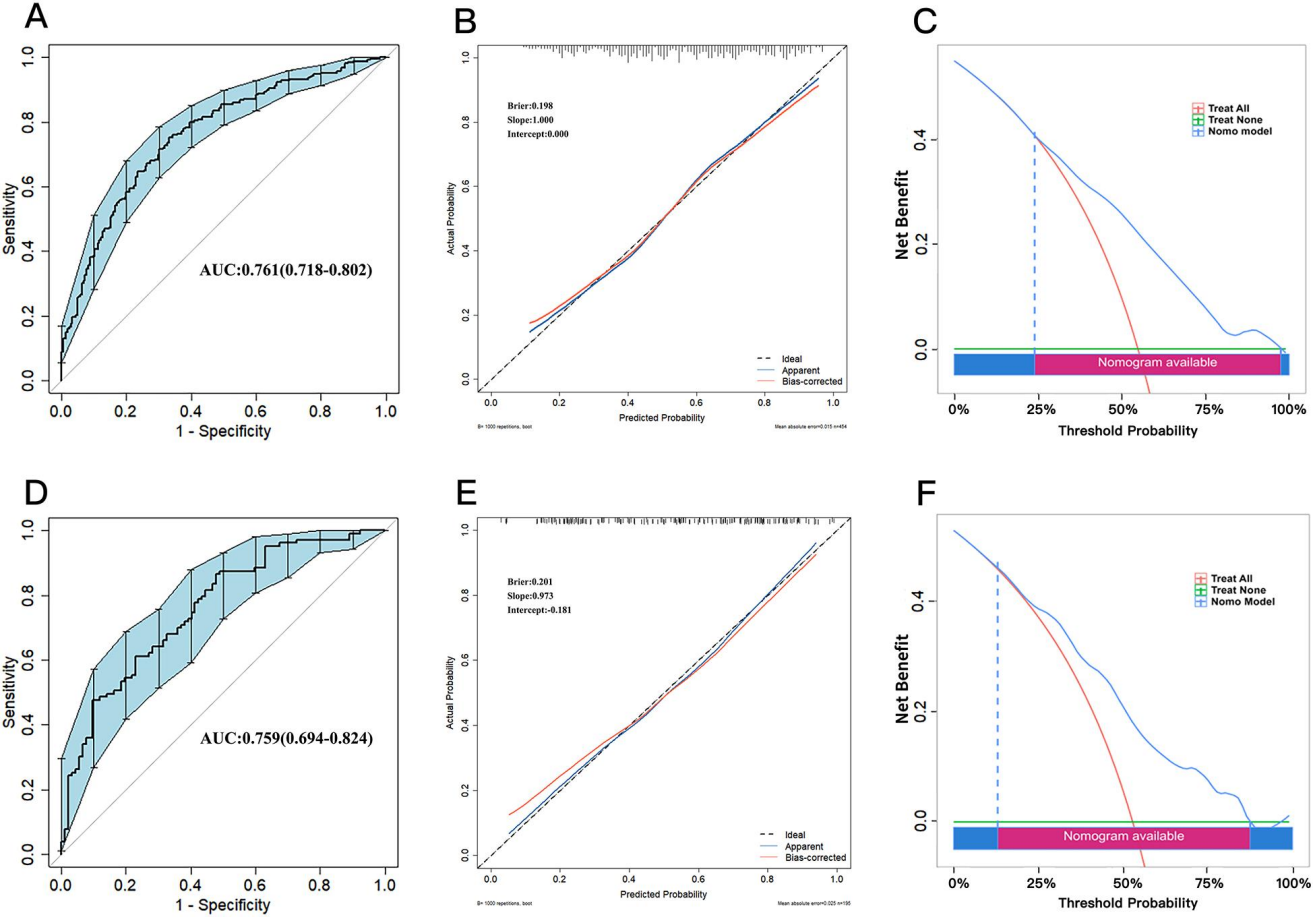
**(A–C)** are the ROC, calibration curve and DCA of the training set. **(D–F)** are the ROC, calibration curve and DCA of the testing set.

The bootstrap internal validation (1,000 replicates) revealed no detectable optimism (average optimism = 0.000). The optimism-correccted AUC was 0.761 (95%CI: 0.718–0.802) (Figure 6A). The model's performance was further confirmed on the validation set, yielding an AUC of 0.759 (95%CI: 0.694–0.824) (Figure 6D). These results indicate that the model possesses strong generalization ability without evident overfitting. The bootstrap-corrected calibration curves for both the training (Figure 6B) and validation sets (Figure 6E) demonstrated good agreement with the ideal line. Quantitatively, the model achieved Brier scores of 0.198 (training) and 0.201 (validation), indicating accurate and well-calibrated predictions. Furthermore, the DCA demonstrated that in the training (Figure 6C) and validation sets (Figure 6F), the nomogram provided a substantial net clinical benefit across a wide range of threshold probabilities.

### 3.4 Evaluation of the rationality of the CHD prediction model

**Figure 7:**
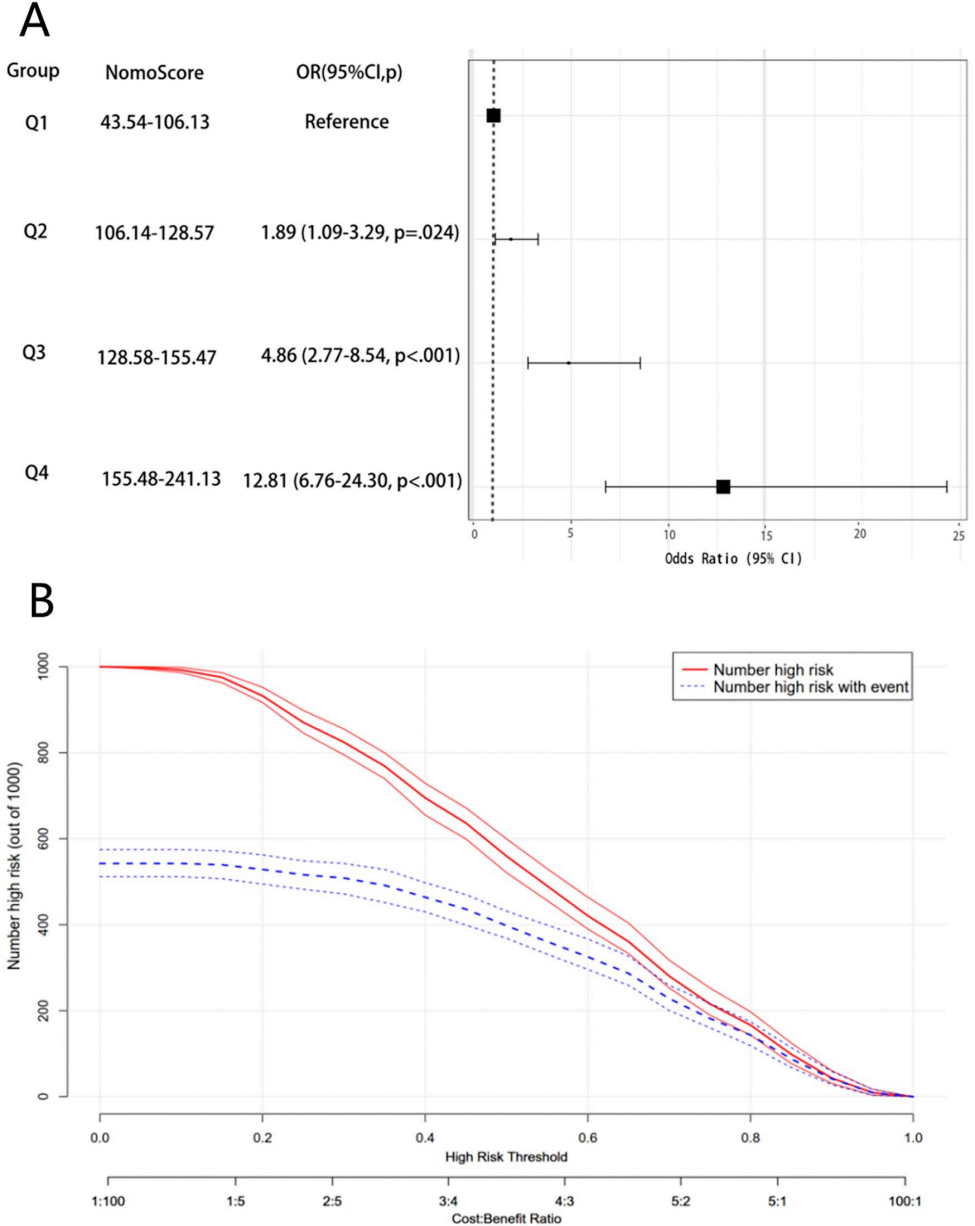
The rationality analysis of the nomogram model. **(A)** Association between the total nomoscore and CHD. **(B)** The CIC of the nomogram model.

The total nomo score for each patient was calculated, and then the total nomo score for all patients was divided into four groups by interquartile spacing (Figure 7A). As illustrated in the figure, CHD risk increases with the increase of the total nomo score. Patients with high quartiles (total score: 155.48–241.13) had a higher CHD risk (OR: 12.81, 95% CI: 6.76–24.30) than those in low quartiles (total score: 43.54–106.13) (*p* < 0.05).

The CIC (Figure 7B) also illustrated that the model successfully predicted high-risk groups of CHD, and when the threshold was greater than 0.6, the prediction probability of the model was in good agreement with the actual probability, indicating that the model had a high clinical prediction efficiency.

## 4 Discussion

Cardiovascular disease has become a major public health problem (1–3), and prevention and treatment of cardiovascular disease are of great significance in improving the life quality of residents and reducing the social burden. Accurate and effective risk assessment is the premise of disease screening, prevention, and treatment. At present, the clinical non-invasive risk assessment of CHD is mainly based on clinical history and laboratory examination and lacks imaging data support (24–26). However, CHD is a complex, multi-factor chronic disease, and a wider range of effective influencing factors must be included to improve the prediction efficiency, and imaging data support is indispensable. The nomograph is an intuitive and comprehensive predictive tool that can predict the probability, risk, and disease prognosis by combining multiple risk factors and imaging indicators in the clinical setting (25). Previous studies (8–11) have exhibited that the carotid can be used as a window to observe coronary artery disease. Therefore, this study is based on CDU to construct a non-invasive risk prediction model for CHD.

The results demonstrated that patient gender, chest pain, diabetes, TG, Apo A, PS, and carotid stenosis were closely related to CHD risk. Using these metrics, we further constructed a predictive model that exhibited high diagnostic performance in both the training and the validation sets (AUC = 0.761 and 0.759, respectively). The Brier scores of the calibration curve on training and the verification sets were 0.198 and 0.201, respectively, indicating that the model had a high consistency. The DCA exhibited that the model had a wide application range and a relatively high clinical net benefit. The CIC also revealed that the model had a high clinical prediction efficiency.

Previous studies on CHD prediction were mostly based on the medical history of patients and risk factors and concluded that males, hypertension, hyperlipidemia, and diabetes were all closely related to CHD (25), and our findings were consistent with them. The difference was that in our study, hypertension was statistically significant only in the univariate analysis but not in the multivariate analysis. This difference may occur because, in this study, hypertension was defined based on the medical history of the patient or the blood pressure measured at admission, and the hypertension degree was not further classified. Additionally, most previous studies have indicated that dyslipidemia is correlated with CHD progression or prognosis (25, 27). In this study, lipid indexes were refined, and univariate analysis found that HDL-C and Apo A were negatively correlated with CHD risk, while TG was positively correlated with CHD risk. After multi-factor analysis, only TG and Apo A were independent predictors of CHD. The analysis was performed because the relationship between HDL-C and Apo A is close and complex, and the association between Apo A and CHD may be stronger than that of HDL-C. The mechanism between the two needs further research and exploration in the future.

Previous studies have used CDU to measure plaque parameters to assist clinicians in predicting cardiovascular events (7, 13, 28, 29). However, because plaque measurement and evaluation are relatively cumbersome and require professional sonographers, this study adopted the Rotterdam method recommended by the American Society of Echocardiography to calculate PS (16), which is simple and fast and can be quickly obtained by non-sonographers. Previous studies have compared PS with MPH and TPA and found similar results in predicting one-year major adverse cardiovascular events (17). As mentioned above, MPH and TPA require additional measurement and calculation, while PS is easier to obtain and can be quickly completed at the bedside. Therefore, this study adopted PS as an indicator and found that it is closely related to CHD risk, which can assist clinicians in carrying out effective risk assessment for patients.

Previous studies have found that patients with carotid stenosis also have a higher prevalence of CHD (11), and carotid stenosis can predict CHD prognosis (30, 31). Our study also illustrated that carotid stenosis was an independent predictor of CHD (OR: 1.817, 95% CI: 1.052–3.205), consistent with previous findings.

The mechanism of plaque formation is that the vascular endothelium is damaged, leading to lipid deposition, which in turn triggers chronic inflammatory responses and eventually forms sclerotic plaques on the arterial vessel walls. Therefore, some scholars (32) have utilized machine learning models and relied on routine physical examinations and blood biochemical indicators to predict the occurrence of carotid artery plaques. Some scholars (33) have also pointed out that machine learning models established based on non-contrast cardiac gated tomography and clinical data can effectively predict CHD. Our research concept is the same as theirs, and it is also based on the most basic clinical data. The difference is that we rely on carotid artery ultrasound examinations. Arteriosclerosis is a systemic process, and atherosclerosis of the carotid artery and coronary artery often occur concurrently. Carotid artery lesions can be relatively easily obtained through carotid artery ultrasound, while coronary artery lesions mainly rely on CCTA and CAG. These methods involve radiation exposure, are relatively expensive, and have more complex operations. Therefore, we utilized carotid ultrasound in combination with other clinically relevant factors of patients to predict coronary artery lesions, with the aim of establishing a non-invasive and data-accessible model for the early assessment of CHD risk.

In summary, we constructed a prediction model of CHD based on CDU, which is simple and easy to use and can provide an effective risk assessment for patients. Despite the advantages of this study, there are some limitations. First, the definition of CHD was mainly based on anatomical stenosis, and was defined as ≥50% stenosis in any major coronary artery, without considering functional significance(such as fractional flow reserve). Future research combined with functional assessment will help to define the affected groups more accurately. Second, this study was a retrospective single-center clinical study, all participants were hospitalized patients with suspected CHD, which introduces potential selection bias. So the findings cannot be generalized to the general population or outpatient screening settings. In the future, we will continue to study this group of people and incorporate more variables, such as behavioral and psychological factors, to further verify and improve the model performance. Finally, we will conduct external verification of external data sets in future work.

## 5 Conclusion

This study constructed a simple and easy-to-use nomo prediction model for CHD patients based on CDU, which has high diagnostic performance, calibration, and clinical value. Junior doctors only need to quickly understand PS and carotid stenosis through CDU, and then, combined with patient clinical indicators, they can quickly and effectively predict CHD risk to provide personalized medical support for patients.

## Data Availability

The raw data supporting the conclusions of this article will be made available by the authors, without undue reservation.
